# Advancing Bongkrekic Acid Detection: From Conventional Instrumental Analysis to Advanced Biosensing for Cross-Toxin Applications

**DOI:** 10.3390/foods15030476

**Published:** 2026-01-30

**Authors:** Zhen Chen, Danni He, Wenhan Yu, Xianshu Fu, Lingling Zhang, Mingzhou Zhang, Xiaoping Yu, Zihong Ye

**Affiliations:** 1 Key Laboratory of Microbiological Metrology, Measurement & Bio-Product Quality Security, State Administration for Market Regulation, College of Life Sciences, China Jiliang University, Hangzhou 310018, China; cz18867121109@163.com (Z.C.); 15215965903@163.com (D.H.); ywh1835628626@163.com (W.Y.); zmzcjlu@cjlu.edu.cn (M.Z.); yxp@cjlu.edu.cn (X.Y.); zhye@cjlu.edu.cn (Z.Y.); 2 Linping District Center for Disease Control and Prevention of Hangzhou City, Hangzhou 311199, China

**Keywords:** bongkrekic acid, structurally analogous toxins, biosensors, immunoassays, food safety

## Abstract

Bongkrekic acid (BKA), a highly lethal toxin, has been implicated in frequent poisoning incidents in recent years, posing a serious threat to global food safety and creating an urgent need for rapid and sensitive detection methods. This review provides a systematic analysis of the entire BKA detection technologies, covering sample pretreatment techniques, instrumental analysis, immunoassays, and biosensing methods. It assesses the merits of key methods and also explores the strategic cross-application of detection paradigms developed for analogous toxins. This review delivers a comprehensive and critical evaluation of BKA detection technologies. First, it discusses sample pretreatment strategies, notably solid-phase extraction (SPE) and QuEChERS. Subsequently, it analyzes the principles, performance, and applications of core detection methods, including high-performance liquid chromatography–tandem mass spectrometry (HPLC-MS/MS), high-resolution mass spectrometry (HRMS), time-resolved fluorescence immunoassay (TRFIA), dual-mode immunosensors and nanomaterial-based sensors. Instrumental methods (e.g., HRMS) offer unmatched sensitivity [with a limit of detection (LOD) as low as 0.01 μg/kg], yet remain costly and laboratory-dependent. Immunoassay and biosensor approaches (TRFIA and dual-mode sensors) enable rapid on-site detection with high sensitivity (ng/mL to pg/mL), though challenges in stability and specificity remain. Looking forward, the development of next-generation BKA detection could be accelerated by cross-applying cutting-edge strategies proven for toxins—such as Fumonisin B1 (FB1), Ochratoxin A (OTA), and Aflatoxin B1 (AFB1)—including nanobody technology, CRISPR-Cas-mediated signal amplification, and multimodal integrated platforms. To translate this potential into practical tools, future research should prioritize the synthesis of high-specificity recognition elements, innovative signal amplification strategies, and integrated portable devices, aiming to establish end-to-end biosensing systems capable of on-site rapid detection through multitechnology integration.

## 1. Introduction

**Figure 1:**
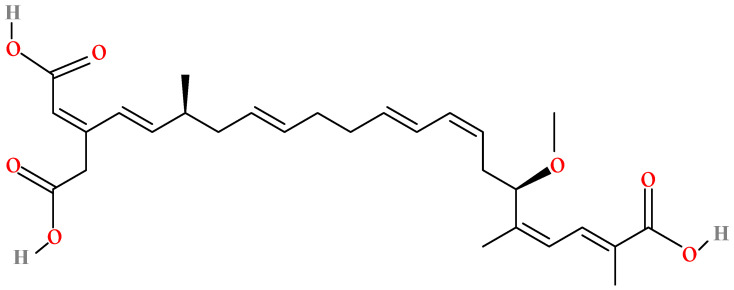
BKA two-dimensional chemical structural formula.

**Figure 2:**
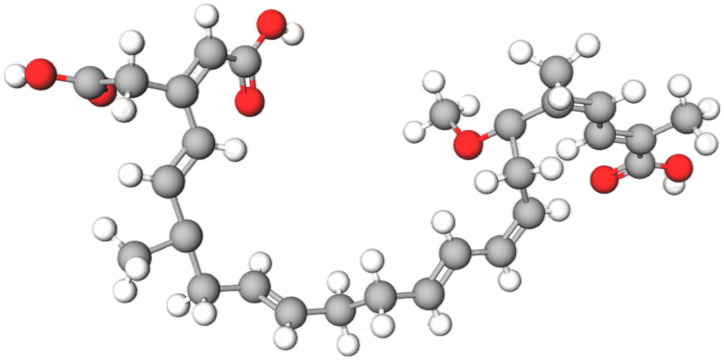
BKA Three-Dimensional Ball-and-Socket Model.

Bongkrekic acid (BKA), a formidable foodborne toxin produced by *Burkholderia gladioli* (formerly classified as *Pseudomonas cocovenenans* subsp. *farinofermentans*), is characterized by its high potency and association with specific fermented and fungal commodities [1]. With its initial report dating back to contaminated coconut products in Indonesia in 1934 [2], BKA continues to be a significant hazard in foods such as fermented rice, black fungus (*Auricularia auricula*), tremella (*Tremella fuciformis*), and similar commodities. The molecular structure of BKA (C_28_H_38_O_7_) was elucidated in 1960 [3]. Its two-dimensional chemical structure and three-dimensional ball-and-stick model are presented from the molview website in Figure 1 and Figure 2, respectively. With a molecular weight of approximately 486.6 g/mol, BKA features a conjugated polyene system within its 22-carbon backbone. The tricarboxylic acid architecture is defined by carboxyl groups (-COOH) at positions C-3 and C-28. The molecule is further decorated with a 17-methoxy group (-OCH_3_), multiple methyl (-CH_3_) substituents, and a carboxymethyl (-CH_2_COOH) side chain. These functional groups collectively contribute to its high lipophilicity and bioactivity.

Purified BKA is an amorphous white solid with good solubility in organic solvents and alkaline solutions and a pronounced affinity for activated carbon. While BKA exhibits high structural and thermal stability, it is susceptible to degradation under acidic conditions and ultraviolet irradiation [4,5].

BKA exerts its potent toxicity by competitively inhibiting the mitochondrial ADP/ATP carrier, inhibiting cellular energy metabolism [6]. Initial clinical symptoms include gastrointestinal and neurological disturbances. As the toxin disseminates, it can induce congestion, edema, and necrosis in major organs such as the lungs, liver, heart, brain, and kidneys. In severe cases, this culminates in hepatic coma, central nervous paralysis, and fatal respiratory failure due to respiratory failure. The lethality of BKA poisoning, for which no specific antidote exists [7], results primarily from multi-organ failure. The median lethal dose (LD_50_) of BKA is a critical parameter for assessing its acute toxicity. Animal studies indicate that the oral LD_50_ in mice ranges from 0.68 to 6.84 mg/kg, while the intravenous LD_50_ is 1.41 mg/kg [8], demonstrating its high potency. Additional research suggests that blood concentrations of 200–300 μg/L have proved lethal. Extrapolating from the lethal blood concentration and a standard human blood volume (4.2–4.8 L), the estimated lethal oral dose is approximately 0.8–1.5 mg, further confirming the toxin’s extremely low lethal threshold [3]. Consequently, there is no acceptable daily intake (ADI), as any detectable exposure is considered potentially hazardous. This reality heightens the urgency for developing highly sensitive detection technologies.

BKA ranks among the most lethal toxins, with a mortality rate ranging from 40% to 100% [9]. As systematically summarized in Table 1, documented BKA poisoning incidents span 130 years (1895–2025) across multiple regions. While the toxin has long been endemic to China and Southeast Asia, with the earliest and most concentrated outbreaks recorded in Java and Indonesia, its persistence in China has continued into recent years (2023–2025). A 2024 North American case further highlights that BKA poisoning is no longer confined to tropical and subtropical regions. Rather, the global distribution of traditionally fermented foods has elevated it to a potential public health concern with worldwide implications. Currently, global regulation of BKA lacks a unified detection and oversight framework, exhibiting marked regional variations. Incidents of BKA poisoning are predominantly concentrated in a few countries, including China. China has established specific testing standards, with GB 7096-2014 [10] stipulating a maximum limit of 0.25 mg/kg for BKA in silver ear mushrooms and their products. GB 5009.189-2023 provides a complementary LC-MS/MS instrumental detection method, extending the range of sample matrices to include cereals, edible fungi, rice noodles, and other food categories. In contrast, Europe, America, Japan, and South Korea have not yet included BKA in mandatory monitoring lists due to the rarity of BKA poisoning incidents in these regions. Countries such as Indonesia and Mozambique, though historically high-incidence zones, currently lack standardized testing methods, with only a handful of researchers employing laboratory LC-MS/MS or HRMS methods. Overall, the global regulatory landscape for BKA can be summarized as follows: in-depth research and mandatory controls exist in specific high-risk regions (China), while the vast majority of countries worldwide exhibit monitoring gaps, with severely inadequate detection capabilities.

BKA contamination does not induce noticeable changes in the odor or color of affected foods, and it can occur in a wide range of food products, posing a significant risk to food safety. Given the severe impact of BKA poisoning on human health, there is an urgent need to develop efficient, rapid, and highly sensitive detection methods to minimize potential risks. Reviews on BKA over the past two years have primarily focused on phased summaries of the toxin’s contamination distribution in food matrices, its toxicological mechanisms, traditional detection methods, and emerging technologies. Compared with existing reviews, this paper not only comprehensively outlines the entire technical workflow for BKA detection, from sample preparation to analysis, but also comparatively evaluates current detection technologies across various dimensions, including sensitivity, detection time, cost, and field applicability. This aims to demonstrate the advantages of each detection method and its suitability for different scenarios. Furthermore, this paper introduces for the first time the concept of a detection technology transfer framework based on structurally similar toxins. By systematically analyzing the similar structural characteristics (molecular size, chemical properties, key functional groups, and carbon chain structures) between BKA and Fumonisin B1 (FB1), ochratoxin A (OTA), and aflatoxin B1 (AFB1), it summarizes the established rapid detection technologies for these three toxins for transferable application in the rapid detection of BKA. Finally, from a technology integration perspective, this paper proposes that future BKA detection technology development should focus on integrating advanced nanomaterials, innovative signal amplification strategies, and portable devices to establish a comprehensive on-site rapid detection system. Distinct from traditional reviews emphasizing summaries of existing technologies, this paper aims to provide more practically relevant recommendations for the BKA detection field through a multidimensional innovative perspective.

## 2. Sample Preparation Techniques for BKA in Complex Matrices

**Figure 3:**
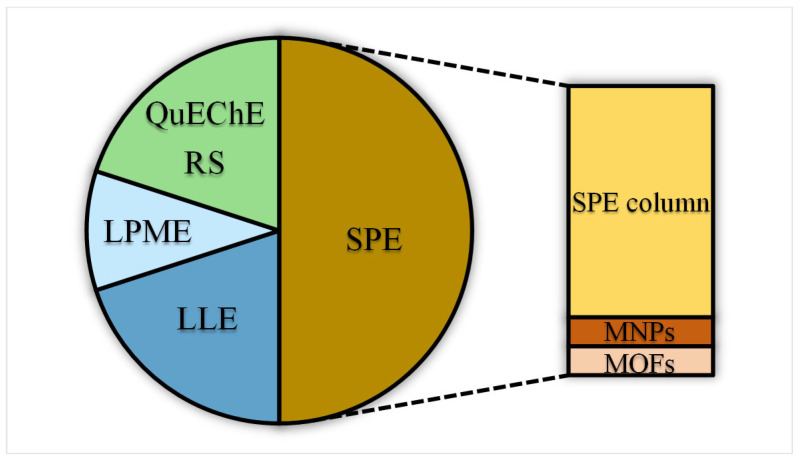
Pie chart showing the proportion of pre-treatment purification technology for BKA.

Sample preparation constitutes a crucial step in the analysis of BKA, as the recovery rate directly affects the reliability of the test results. BKA-containing samples are characterized by complex matrices with high levels of proteins, polysaccharides, lipids, and pigments. These components not only increase background noise and reduce column separation efficiency, but also readily lead to tubing blockages, column contamination, and detector fouling, thereby causing peak distortion and retention time shifts—a phenomenon commonly known as the “matrix effect” [23]. Consequently, rigorous sample pretreatment is indispensable for mitigating matrix interference. Figure 3 illustrates the current distribution of pretreatment techniques used in the detection of BKA. The two pie charts provide a comprehensive overview of these technologies. Solid-phase extraction (SPE) columns, along with their derived and advanced forms (magnetic nanoparticles, MNPs and metal–organic frameworks, MOFs), undoubtedly represent the dominant approaches. Conventional SPE remains the most widely used method due to its established reliability, while the QuEChERS technique also occupies a considerable proportion owing to its rapidity and efficiency. (Micro) Liquid–liquid extraction ((M)LLE) continues to be used consistently. Although emerging adsorbent materials currently represent a relatively small proportion, they indicate important directions for future development.

### 2.1. Extraction Techniques

Current mainstream extraction techniques for BKA include vortex oscillation, shaking extraction, ultrasonic extraction, microwave-assisted extraction, high-speed homogenization [24], and mechanical shaking [25]. Extraction strategies are tailored to sample matrix properties, ranging from single to combined methods. Among rapid techniques, vortex oscillation is particularly effective for its low solvent consumption (<50 mL) and short processing period (2–5 min). A representative application by Zhang et al. [26] achieved excellent recoveries (88.7–101.5%) for BKA in fermented rice flour, demonstrating its high efficiency and robustness. Ultrasonic extraction significantly improves mass transfer efficiency between phases through cavitation and mechanical vibration. Xu et al. [27] reported an average recovery rate of 104% after one hour of ultrasonic treatment when extracting BKA from rice noodle samples. This method also simplifies sample preparation by allowing one-step purification with acetonitrile, while the ultrasonication step enhances the overall extraction speed.

### 2.2. Liquid–Liquid Extraction (LLE)

LLE is a classical separation and purification technique based on the difference in distribution coefficients of the target compound and matrix components between two immiscible phases, typically aqueous and organic [28]. In practical applications, solvents such as dichloromethane, methanol, acetonitrile, and low-concentration acidic mixtures have shown favorable extraction efficiency for BKA. For instance, Xu et al. [29] employed a methanol–dichloromethane biphasic system combined with phosphoric acid–adjusted dispersion extraction to isolate BKA from plasma. Zhong et al. [30] reported that methanol–ammonia solutions effectively extracted the toxin from rice flour and corn flour, although this system was less suitable for high-sugar substrates such as silver ear fungus. For matrices rich in polysaccharides and proteins, Wen et al. [31] recommended the addition of anhydrous ammonium sulfate or magnesium sulfate during LLE to precipitate impurities, thereby reducing the risk of column blockage and improving extraction efficiency. Luo et al. [32] utilized an ultrasonic-assisted liquid–liquid extraction method to purify BKA from rice noodles and rice vermicelli. This approach not only accelerated the extraction process and simplified sample preparation but also achieved satisfactory spike recovery rates ranging from 81.0% to 90.4%.

### 2.3. Liquid Phase Micro-Extraction (LPME)

LPME is a miniaturized alternative to conventional LLE. It achieves selective distribution of the target analyte between immiscible phases by adjusting physicochemical parameters such as PH, temperature, and ionic strength. By contrast, this method greatly reduces the consumption of organic solvent—often to the microliter level—thereby aligning with the principles of green chemistry [33]. Huang et al. [34] employed floating organic droplet solidification liquid-phase microextraction (FLSPME) in their investigation of BKA detection, representing a quintessential application of liquid-phase microextraction techniques. This study selected dodecyl alcohol, a low-density and low-melting-point compound, as the extractant to form solidifiable suspended droplets. The optimal extraction conditions were optimized and determined as 30 μL of dodecyl alcohol and 15 min of extraction at room temperature, achieving spiked recovery rates of 83.5% to 98.6%.

### 2.4. Solid Phase Extraction (SPE)

SPE is the most extensively used pretreatment method for detecting BKA in rice, both domestically and internationally. The principle of SPE relies on the selective adsorption of target compounds onto solid adsorbents, which separates them from the sample matrix and interfering substances. Interfering components are then washed away using solvents of different polarities, and the target compounds are subsequently desorbed with an appropriate solvent, achieving effective separation and enrichment. This method offers several advantages, such as high recovery, short processing time, broad compatibility, low environmental impact, and resistance to emulsification [35]. SPE is commonly categorized according to the type of adsorbent used.

#### 2.4.1. Solid-Phase Extraction Column

The chemical structure of BKA includes multiple free carboxyl groups, which leads to very low retention on conventional reverse-phase (C18) and hydrophilic interaction liquid chromatography (silica, HLB) extraction columns [36]. Commercially available extraction columns are mainly based on mixed-mode weak anion exchange and mixed-mode strong anion exchange materials. Qin, Wang, Lin, Su et al. [37,38,39,40,41] employed a method combining solvent extraction and solid-phase extraction with mixed-mode strong anion exchange columns to analyze various sample matrices such as snail noodles, Jianqu, Liushengqu, and red yeast rice. Their studies indicated that mixed-mode strong anion exchange mini-columns provided better extraction efficiency than pure strong anion exchange columns [42]. In addition, researchers and companies have developed extraction columns with different sorbent materials designed according to the properties of the target compounds. For instance, Waters Corporation introduced novel PRIME solid-phase extraction columns that enable purification of multiple toxins from complex matrices. Using a three-step procedure—loading, rinsing, and elution—these columns eliminate the need for pretreatment and equilibration steps. Hu et al. [43] were the first to apply Oasis PRIME HLB extraction columns for the simultaneous detection of five toxins: aflatoxins B1, B2, G1, G2, and BKA, achieving satisfactory recovery rates ranging from 80.5% to 106.6%. Subsequently, Fang et al. [44] used the newer Oasis PRiME HLB 6cc extraction cartridge coupled with a PriboFast MFC 336 purification column to simultaneously determine BKA, zearalenol, and fumonisins in edible fungi and rice noodles, with recoveries between 83% and 96%. Furthermore, composite purification columns that incorporate polar, non-polar, and ion-exchange mixed media can selectively adsorb non-target impurities from samples. Meng et al. [45] employed a PriboFast MFC335 multifunctional column, which simplified the traditional solid-phase extraction process by omitting steps such as activation, rinsing, and elution. Sample purification was achieved with a single pass through the column, showing high practical utility and development potential. However, a fundamental challenge remains in solid-phase extraction for BKA detection: the general-purpose adsorbents (graphitized carbon black, GCB; primary secondary amine, PSA; octadecylsilane, C18) are not well suited to the unique physicochemical properties of BKA, which is both lipophilic and contains multiple carboxyl groups. This highlights the need to develop novel adsorbents specifically designed for the chemical characteristics of BKA.

#### 2.4.2. Magnetic Solid-Phase Extraction (MSPE)

MSPE is an innovative sample preparation technique that employs magnetic nanomaterials as the core material. Its basic principle involves the use of functionalized magnetic nanoparticles, such as Fe_3_O_4_, as adsorbents. Due to their high specific surface area, superparamagnetic behavior, and easily modifiable surfaces, these nanoparticles can effectively concentrate trace amounts of toxins from complex sample matrices. During the extraction process, the magnetic nanoparticles are dispersed into the sample solution, where they interact with target toxins through surface functional groups (-OH, -COOH, specific ligands). The particles are then rapidly separated using an external magnetic field, significantly reducing both organic solvent consumption and sample processing time. In a study by Liang et al. [46], alkaline-modified heulandite nanotubes (HNTs) were used together with Fe_3_O_4_ nanoparticles, synthesized via co-precipitation, to produce Fe_3_O_4_/HNTs magnetic composites for MSPE. The hydroxyl groups on the modified HNTs bind specifically to the carboxyl groups of BKA and its isomer, isobongkrekic acid (IBKA), allowing efficient and direct magnetic extraction of these compounds. The method achieved a quantification limit of 1.0 μg/kg and recoveries of 79.8–102.6%. However, the preparation of such magnetic adsorbents is associated with high cost, high energy consumption, and difficulties in maintaining consistency between batches, which currently hinders large-scale commercial production.

#### 2.4.3. Metal–Organic Framework Materials (MOFs)

MOFs are emerging porous materials formed through the coordination of metal ions with organic ligands. They exhibit tunable pore structures, high specific surface areas, and numerous active sites, allowing efficient enrichment and selective adsorption of various toxins. MOFs combine physical adsorption mechanisms, such as van der Waals forces and capillary action, with chemical interactions including coordination and electrostatic effects. Their high thermal stability and recyclability further support practical application. Fang et al. [47] developed a dispersive solid-phase extraction method using a nitro-modified zirconium-based MOF (NO_2_-MOF) for efficient extraction of BKA from wood ear fungus and rice noodles. The nitro-functionalized Zr-MOF was synthesized via a thermal solvent method. The nitro groups on the material interact with BKA through multiple mechanisms, including electrostatic attraction, π–π stacking, and hydrogen bonding, which together enable highly selective adsorption and controllable desorption. This method achieved a limit of quantification (LOQ) of 1.6 μg/kg, a limit of detection (LOD) of 0.5 μg/kg, and spike recoveries of 75–96%. Nevertheless, selectivity was limited. It strongly adsorbed tetrodotoxin but did not allow its elution, while failing to adsorb other non-target compounds. This behavior suggests a risk of both false positives (tetrodotoxin competing for binding sites intended for BKA) and false negatives (lack of interference from tetrodotoxin but potential interference from other common anions not yet validated). In complex food matrices, such limited selectivity could lead to significant variability in recovery rates.

### 2.5. QuEChERS (Quick Easy Cheap Effective Rugged Safe)

QuEChERS, introduced by Anastassiades in 2003, is now widely used for the analysis of diverse complex matrices such as foods, biological fluids, and environmental samples [48]. This approach consists of two main steps: salting-out and dispersive solid-phase extraction (dSPE). It uses extraction salts (e.g., magnesium sulfate and sodium salts) to induce a salting-out effect, separating target analytes from the sample matrix and transferring them into an acetonitrile phase. Subsequent cleanup is carried out using porous adsorbents (GCB, PSA, and C18) along with additional salts, making the method environmentally friendly, rapid, and simple to perform [49,50,51,52]. Liang et al. [53] applied a modified QuEChERS approach combined with UPLC-Q-Orbitrap HRMS to determine BKA in rice noodles, obtaining recovery rates between 90.6% and 96.8%. In another study, Zou et al. [54] reported that during the modified QuEChERS cleanup of oryzanol in silver ear fungus and wood ear fungus, GCB effectively removed pigments and other interferences from silver ear extracts. However, it also adsorbed the hydrophobic BKA, leading to reduced recovery. Lu et al. [55] optimized QuEChERS conditions for rice paste extracts, recommending the use of 200 mg C18 and 900 mg of anhydrous magnesium sulfate. Lu et al. [56] compared four adsorbents—C18, PSA, C18 + PSA, and dSPE EMR-Lipid—for purifying BKA in Hanzhong noodle sheets. The results showed that dSPE EMR-Lipid provided significantly higher recoveries (86.7–90.0%) than the other three materials, which all resulted in recoveries below 50%. Currently, there is no international standard QuEChERS procedure for BKA. The method modifications employed across different studies vary substantially, leading to poor comparability of results and challenges in meeting trace-level regulatory requirements.

## 3. Detection Methods for BKA

In recent years, significant progress has been achieved in the development of Detection Methods for BKA. Based on core detection principles, technological platforms, signal conversion mechanisms, and application scenarios, the methods can be broadly classified into three categories:

- Instrumental Analytical Methods (e.g., chromatography–UV detection) Separate and quantify toxins based on physicochemical properties such as chromatographic behavior and ultraviolet absorption.
- Immunological Rapid Detection Methods (e.g., ELISA, colloidal gold test) rely on specific antigen–antibody binding, often coupled with labeled colorimetric detection.
- Biosensor Rapid Detection Techniques (e.g., electrochemical/optical biosensors) Integrate biological recognition elements (antibodies, aptamers, or enzymes) with signal transduction technologies for real-time monitoring.

### 3.1. Instrumental Analytical Methods

In early instrumental analytical methods, thin-layer chromatography and ultraviolet spectrophotometry were frequently utilized as detection techniques. Early Thin-layer chromatography (TLC) [57] suffers from limited separation efficiency due to the properties of the stationary phase, coupled with poor quantitative precision. Ultraviolet spectrophotometry, meanwhile, is hampered by significant matrix interference, poor selectivity, and low sensitivity. These limitations have led to their gradual replacement by newer instrumental analytical technologies. It is noteworthy that modern high-performance thin-layer chromatography (HPTLC) has seen significant performance enhancements through the use of finer stationary phases and fully automated operation. Particularly when coupled with mass spectrometry detectors, most notably in the integrated high-performance thin-layer chromatography with electrospray ionization tandem mass spectrometry (HPTLC-ESI-MS/MS) platform. The technique has evolved into a robust analytical tool, offering unique advantages for applications in drug and toxin analysis. Regarding trace toxin detection, data cited by Feng [58] indicate that HPTLC-MS achieves a LOD of 0.52 μg/kg for AFB1 in peanuts, rivaling the sensitivity of high-performance liquid chromatography–mass spectrometry (HPLC-MS) methods. However, for the specific toxin BKA, no literature has yet validated or supported the HPTLC-ESI-MS/MS method. Contemporary instrumental analytical detection methods primarily employ high-performance liquid chromatography (HPLC) and high-performance liquid chromatography–mass spectrometry (HPLC-MS).

HPLC relies on differences in the distribution coefficients of components between the stationary and mobile phases. By modulating the composition and flow rate of the mobile phase, target molecules are separated sequentially within the chromatographic column, followed by qualitative and quantitative analysis using high-sensitivity detectors. While the latest Chinese national standard (GB 5009.189-2023) designates HPLC as the primary detection method, its application is officially recognized only for specific commodities like silver ear fungus and fermented rice flour, highlighting the need for broader standardization [59]. Chen et al. [60] developed an HPLC method with a diode array detector for detecting BKA in multiple rice-fermented foods, including cereals, tubers, and wet rice noodles. Kang et al. [61] identified the PH of the mobile phase as a critical factor affecting chromatographic behavior, showing that acidic mobile phases containing formic or acetic acid significantly improve peak shape. Yu et al. [62] reported that increasing the injection volume within the range of 10–30 µL resulted in poor peak shape and noticeable tailing of the target compound. Traditional HPLC methods often involve long analysis times, are susceptible to matrix interference, and may show considerable qualitative and quantitative inaccuracies. They are particularly inadequate for samples with low analyte concentrations, frequently demand large sample volumes, and thus fall short of meeting current requirements for high-sensitivity toxin detection.

Compared to HPLC, HPLC–MS offers a simpler procedure, shorter analysis time, higher specificity, and improved sensitivity. The latest Chinese national standard (GB 5009.189-2023) has incorporated LC–MS/MS, achieving a detection limit of 1.0 μg/kg and a quantification limit of 3.0 μg/kg. This standard has also expanded its scope from silver ear fungus and fermented rice-based products to include black fungus and cereal products, thereby covering high-risk food matrices associated with recent BKA poisoning incidents. In recent years, several new HPLC-based methods have been developed to address sensitivity and matrix interference challenges in BKA detection. Zeng et al. [63] established an ultra-high performance liquid chromatography–mass spectrometry (UPLC–MS) method for detecting BKA in rice noodles and rice vermicelli, achieving significantly improved sensitivity with detection and quantification limits of 0.1 μg/kg and 0.2 μg/kg, respectively. Zhang et al. [64] applied high-performance liquid chromatography coupled with triple quadrupole tandem mass spectrometry (HPLC-triple quadrupole tandem mass spectrometry, HPLC–QQQ-MS/MS) for the simultaneous detection of BKA and IBKA in plasma and urine. This method reached a detection limit of 0.02 μg/L and a quantification limit of 0.05 μg/L, representing an improvement in sensitivity by several orders of magnitude compared to methods designed for food matrices. It also filled a technical gap in the clinical diagnosis of BKA poisoning. However, a limitation of this approach is the relatively low extraction recovery rate for plasma samples. In response, Fang et al. [65] incorporated stable isotope dilution technology to enable rapid detection of BKA and IBKA in both plasma and urine. Experimental results demonstrated that this technique [66] effectively compensates for matrix effects and minimizes the influence of varying sample matrices on the chromatographic peak response of target compounds, thereby yielding more accurate quantitative results. Similarly, Xia, Li et al. [67,68] developed an isotope internal standard-based method with UPLC–MS/MS for the rapid detection of BKA in multiple food matrices, including white fungus, black fungus, rice, and maize.

In recent years, HPLC–MS has evolved substantially, shifting from traditional low-resolution tandem mass spectrometry to high-resolution mass spectrometry (HRMS). HRMS operates at very high resolution, providing accurate mass measurements with mass accuracy better than 5 ppm. Its principal technical forms of HRMS include time-of-flight mass spectrometry and orbitrap mass spectrometry. Li et al. [69] developed a method using liquid chromatography coupled with time-of-flight mass spectrometry (LC-TOF-MS) to detect BKA in infant rice cereal, milk powder, and cereal powder. Using negative ion mode electrospray ionization and targeted MS^2^ scanning, this method quantified BKA based on characteristic ion mass-to-charge ratios with external calibration, demonstrating strong qualitative performance. Zhou et al. [70] applied quadrupole time-of-flight mass spectrometry (Q-TOF) with a dual-jet electrospray ion source in negative ion mode. Full-scan MS acquisition enabled both qualitative and quantitative determination of BKA in human whole blood. Han et al. [71] used a quadrupole–orbitrap high-resolution mass spectrometer with negative electrospray ionization, performing high-accuracy full MS and MS^2^ scans simultaneously, completing the analysis within 20 min. Zhu et al. [72] employed quadrupole–electrostatic orbitrap high-resolution mass spectrometry to rapidly determine the mycotoxin content in five traditional Chinese medicinal materials (Liu Shen Qu, Jian Qu, Ganoderma lucidum, Poria cocos, and Sargassum), with detection limits ranging from 10 to 20 μg/kg and quantification limits from 30 to 50 μg/kg. Zhao et al. [73] applied quadrupole orbitrap HRMS for highly sensitive detection in multiple matrices—including vomit, glutinous rice balls, rice noodles, and black fungus—achieving a detection limit as low as 0.01 μg/kg. Despite these advancements, the high cost of HRMSinstruments restricts their widespread adoption. To date, no other HRMS methods for BKA have been reported, indicating that this field remains open for further research and development.

Although instrumental detection methods demonstrate exceptional sensitivity and accuracy in BKA detections, they exhibit significant limitations in practical application. In terms of cost, these methods rely on expensive large-scale instruments (such as mass spectrometers) and specialized operators, entailing high testing costs and costly maintenance, making widespread adoption in resource-limited settings infeasible. Regarding time, instrumental analysis often requires several hours, resulting in low testing efficiency that fails to meet the rapid screening demands of acute poisoning incidents. Regarding reproducibility, significant variations exist across laboratories in chromatographic column models, mass spectrometry parameter settings, and sample preparation protocols, leading to poor data comparability. For instance, in LC-MS methods, variations in mobile phase ratios and ion source conditions across laboratories can yield relative deviations of 10–20% in results for identical batches of BKA-positive samples. Regarding matrix effects, proteins, polysaccharides, and lipids in complex food matrices (such as high-sugar silver ear fungus or high-fat cereal products) significantly reduce mass spectrometry ionization efficiency. Even isotope internal standardization cannot fully eliminate this issue. Existing instrumental methods are predominantly tailored for food matrix analysis, leaving a gap in methodologies for biological samples (e.g., plasma, urine). Instrumental analytical methods struggle to fulfil rapid screening and early warning requirements during the initial stages of poisoning incidents, proving more suitable for post-incident confirmation and quantification.

### 3.2. Immunological Rapid Detection Methods

Immunological rapid detection methods have become a preferred approach for preventing BKA poisoning and enabling rapid on-site diagnosis, owing to their portability, high efficiency, and user-friendly operation. The core principle of this technology relies on the specific binding reaction between antigens and antibodies. During detection, BKA, as the target antigen, forms a stable immune complex through highly specific recognition by its corresponding antibody. To visualize and quantify this binding event, labeling techniques are commonly employed. Established methods reported in the literature include colloidal gold immunochromatography, indirect competitive enzyme-linked immunosorbent assays (ic-ELISA), and time-resolved fluoroimmunoassays (TRFIA), which enable rapid and accurate qualitative and quantitative analysis of BKA in various samples.

#### 3.2.1. Colloidal Gold Immunoassay (CGIA)

CGIA is a rapid detection technique that uses electrostatic adsorption or covalent conjugation to label antibody–antigen complexes. It is widely regarded as a benchmark method in immunochromatography. Cao et al. [74] synthesized colloidal gold nanoparticles using the trisodium citrate reduction method for the detection of BKA. This approach allows qualitative detection with a visual disappearance threshold at 16.0 μg/kg. The quantitative detection limit was 1.2 μg/kg, and the linear range extended from 1.8 to 7.2 μg/kg. However, the method exhibits certain limitations, primarily due to the tendency of antigens and antibodies to detach from the colloidal gold particles, leading to relatively low stability of the labeled probes.

#### 3.2.2. Indirect Competitive Enzyme-Linked Immunosorbent Assays (ic-ELISA)

Ic-ELISA is an immunological technique based on the specific binding between antigens and antibodies. In this method, the target antigen in the sample competes with a known antigen immobilized on the microplate for binding sites on enzyme-labeled antibodies. A higher concentration of the target antigen leads to increased binding to the enzyme-labeled antibody, thereby reducing the amount of antibody available to bind the immobilized antigen. Given that BKA is a small-molecule toxin with constrained epitopes, a competitive assay format is essential. In their work, Wu et al. [75] adopted a heterologous coating strategy, utilizing IBKA as the coating antigen, in contrast to the conventional use of BKA itself. This strategy lowers the cost of the coating antigen, reduces steric hindrance, and improves antibody binding efficiency. The developed method achieved a detection limit of 0.99 ng/mL, demonstrating high sensitivity and suitability for detecting low concentrations of the antigen in complex samples.

#### 3.2.3. Time-Resolved Fluoroimmunoassays (TRFIAs)

TRFIA is a highly sensitive detection technique that uses fluorescent chelates of lanthanide elements, such as europium (Eu^3+^), terbium (Tb^3+^), and samarium (Sm^3+^), as labels for the detection of antigens or antibodies. These chelates replace traditional signal-generating substances, including enzymes, radioisotopes, fluorophores, and chemiluminescent agents. The key principle of TRFIA is based on the long fluorescence lifetime of lanthanide chelates. Upon light excitation, these compounds emit characteristic fluorescence. By introducing a time delay before signal acquisition, the technique effectively distinguishes the long-lasting fluorescence of lanthanide complexes from short-lived background interference, enabling highly sensitive and specific detection. Zhang et al. [76] developed a quantitative fluorescent immunoassay card for this purpose. An artificial antigen was prepared by conjugating a BKA-specific antibody with bovine serum albumin, which was then coated onto a nitrocellulose membrane as the capture material. A proprietary monoclonal antibody against BKA was labeled with time-resolved fluorescent microspheres and dispensed into microplate wells. The membrane and microspheres were integrated into a test card. This method is straightforward to use and suitable for field applications, although its sensitivity is comparatively low, with a LOD of 4 μg/kg. Chen et al. [77] employed europium microspheres and optimized the probe preparation conditions, identifying an activation PH of 6.0 and a conjugation PH of 8.0 as optimal. This approach achieved a lower detection limit of 0.5 μg/kg. Similarly, Lin et al. [78] designed a rapid and sensitive immunochromatographic test strip using europium microspheres to detect BKA in edible fungi. A notable innovation was the incorporation of an independent dual-probe quality control system, in which the control line (SA-biotin conjugate) was physically separated from the test line. This design ensures result validity by confirming that the control line remains visible even if the test line shows no signal. The method allows very rapid on-site detection with results available within 10 s, and demonstrates high sensitivity, with an IC_50_ of 0.264 ng/mL and a linear range of 0.1–10 ng/mL.

#### 3.2.4. Practical Limitations of Immunological Rapid Detection Methods

Immunological rapid Detection methods offer distinct advantages for field screening, yet present shortcomings in technical maturity and practical application. Regarding specificity, the structural similarity between BKA and IBKA renders existing immunological methods incapable of fully distinguishing between them, readily inducing false-positive results. Concerning stability, antibodies readily inactivate during storage, resulting in a short shelf life for test kits and diminished detection signals under elevated temperatures (>35 °C). Regarding reproducibility, antibody activity varies across different batches, potentially compromising the consistency and repeatability of test results. Concerning accuracy, most immunological detection methods rely on visual observation, enabling only qualitative or semi-quantitative testing, which fails to meet the quantitative requirements for trace regulation. Existing immunological detection methods exhibit significant gaps in their application scope, remaining confined to easily processed food matrices such as rice flour and white fungus. Research on complex matrix effects remains inadequate and requires expansion. Immunological detection methods also lag considerably behind laboratory-based large-scale instrumentation in performance, rendering them suitable only for preliminary on-site screening and processing.

### 3.3. Biosensor Rapid Detection Techniques

Biosensor rapid detection techniques detect targets by combining biomolecular recognition elements (antibodies, nucleic acids, enzymes, or nanomaterials) with signal transduction devices. This approach leverages specific interactions between biological molecules and target analytes, including antigen–antibody binding, nucleic acid hybridization, or enzyme–substrate reactions, to achieve fast, sensitive, and specific detection. The core principle of biosensors lies in the conversion of molecular recognition events into measurable physical or chemical signals, including optical, electrochemical, or piezoelectric responses. These signals are subsequently amplified and processed by the detection system, enabling quantitative or qualitative analysis of the target substance.

**Figure 4:**
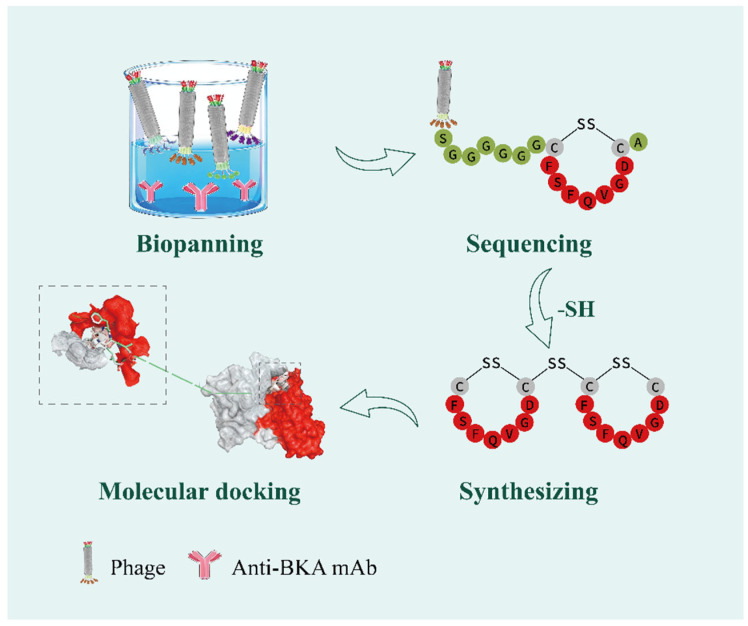
Synthesis of dimer peptidomimetics.

Zhong et al. [79] developed a novel biomolecular recognition element, a molecularly imprinted wooden toothpick (MIPWT) probe, to overcome the limitations of low sensitivity and complicated pretreatment in the detection of BKA. The core of molecular imprinting technology (MIT) lies in its ability to create highly selective recognition sites within a polymer matrix that are specific to target molecules. Molecularly imprinted polymers (MIPs) produced by this method exhibit lock-and-key recognition properties, enabling specific binding to target molecules. MIPs offer predictable structure–activity relationships, excellent stability, high selectivity, and reusability, making them highly suitable for advanced sensor technologies [80,81,82,83]. The key innovations of this work include the adoption of structurally analogous retinoic acid and ricinoleic acid as mixed virtual templates, along with wooden toothpicks serving as substrates, a MIP-coated toothpick integrated design, and the superior characteristics of the toothpick platform. This dual-template approach mimics BKA’s polycarboxylate and unsaturated chain structures, enhancing cavity matching and specificity. With a high-surface-area porous structure, the wooden toothpicks facilitate MIP coating loading, and their tips enable direct voltage application for electrospray ionization (ESI) to eliminate elution steps. The MIP-coated toothpick achieves simultaneous extraction, enrichment, and ionization without chromatographic separation, simplifying sample pretreatment procedures. Furthermore, the toothpick platform boasts low cost, portability, and user-friendliness, rendering it suitable for on-site sampling. Experimental results demonstrated an enrichment factor (EF) of 683 ± 53, a detection limit as low as 0.05 μg/L, and recovery rates ranging from 81% to 97%, which constitutes a major performance improvement and illustrates the entire process of Cao’s design for the detection of BKA [84]. In this study, hybridoma technology was used to produce monoclonal antibodies against BKA. The immunogen BKA-LF and the coated antigen BKA-OVA were synthesized via the activated ester method. After immunizing mice, cell fusion was performed, followed by five rounds of subcloning screening. This process resulted in the establishment of a stable hybridoma cell line named 1B9, which secretes monoclonal antibodies. The purified antibody was identified as subclass IgG1. Evaluation results showed that this monoclonal antibody possesses high affinity (Kd = 0.33 μM), high sensitivity (IC_50_ = 17.9 ng/mL, and strong specificity (cross-reactivity rates below 0.1% against six common mycotoxins, including aflatoxin and T-2 toxin). In the same study, a dual-module immunosensor was developed for the detection of BKA using two independent signal channels: fluorescence and colorimetry. The fluorescence-based module uses red-emission carbon dots (emission at 650 nm) to improve the signal-to-noise ratio, achieving a LOD of 5.7 ng/mL. The colorimetric module is based on the etching of gold nanostructures (AuNSs), producing a visible color change, with an LOD of 8.4 ng/mL. The two detection mechanisms operate independently, allowing cross-verification of results and demonstrating the adaptability of the method for use in various scenarios. Given the high toxicity and instability of BKA, Cao [85] used a synthetically prepared dimeric peptide analog (connected via disulfide bonds) to replace the toxic BKA standard, as shown in Figure 4. This strategy eliminates operational risks and avoids the inefficiency and hazards associated with traditional BKA–carrier protein conjugation in immunoassays. The developed platform supports universal detection with dual-mode output: on-site visual qualitative screening and smartphone-based quantitative analysis. Zhang et al. [86] developed an innovative dual-mode detection platform for BKA using cysteamine-modified gold nanoparticles (CS-AuNPs). The core mechanism relies on electrostatic interactions: at pH = 4, negatively charged tricarboxylic BKA molecules serve as “molecular bridges,” cross-linking positively charged CS-AuNPs and inducing their aggregation. This aggregation causes a red shift in the local surface plasmon resonance (LSPR) absorption peak, leading to a visible color change from burgundy to blue-violet. The ultraviolet–visible (UV-Vis) spectroscopic mode demonstrated high sensitivity, with a detection limit as low as 3.43 nmol/L. Coupled with a 96-well plate design, the system enables efficient high-throughput detection. The smartphone-based colorimetric mode exhibited a slightly higher detection limit but is well-suited for portable point-of-care or home testing. Its portability and rapid response make it highly promising for early clinical screening of BKA poisoning, such as in serum or urine samples. Xuan et al. [87] constructed core–shell structured biomimetic nanospheres (CuS@Au-Pt, CAP) through an efficient self-assembly process. These nanospheres feature a distinctive coral-like rough surface, which significantly enhances colorimetric signal intensity. They also show high antibody affinity (affinity constant Ka = 4.26 × 10^8^ M^−1^, six times greater than conventional gold nanoprobes) and exhibit strong peroxidase-like (POD) activity. The researchers integrated CAP nanospheres as signal markers into a competitive nanozyme-linked immunoassay for detecting BKA. The CAP-NLFIA platform achieved a visual detection limit of 0.5 ng/mL and an instrumental quantification limit of 0.66 ng/mL in colorimetric mode, substantially outperforming conventional gold nanoparticle-based lateral flow immunoassays. Moreover, by leveraging the inherent pseudo-enzymatic activity of CAP to catalyze the TMB–H_2_O_2_ colorimetric reaction, the detection range was nearly doubled, extending from 44 ng/mL to 80 ng/mL. This method also displayed high accuracy in real food samples, including millet flour, corn flour, and white fungus, with spike recovery rates ranging from 80.96% to 119.36%. Lu et al. [88] adapted a high-efficiency flow cytometry-based fluorescent immunoassay platform—originally used in clinical and cellular analysis—to the field of food safety, developing a competitive fluorescent immunosensor for the high-throughput quantification of BKA. This method uses highly specific anti-BKA monoclonal antibodies conjugated to fluorescent magnetic microspheres to form capture probes. When coupled with a flow cytometry detection system, the assay reached a detection limit of 0.56 μg/kg, comparable to that of UPLC–MS/MS, but with a much shorter detection time of under 30 min. Notably, the encoding capability of the microspheres allows for grouping, suggesting potential for future multiplexed detection of various mycotoxins.

Although biosensor rapid detection methods demonstrate considerable development potential, they still fall short in terms of accuracy, reliability, and versatility for quantitative analysis when compared to established large-scale instrumental analytical methods such as LC-MS/MS and HRMS. This necessitates a thorough analytical evaluation. Regarding quantitative accuracy, mass spectrometry techniques represented by HRMS can already achieve absolute quantification at the μg/kg or even pg/kg level, with minimal influence from matrix effects. By contrast, existing biosensor quantification relies on standard curves with narrow linear ranges, and signal response is susceptible to complex sample matrices (PH, ionic strength, impurities). Accuracy and repeatability at trace toxin concentrations fall significantly short of mass spectrometry. Regarding practicality, established LC-MS/MS and HRMS systems have well-defined standard operating procedures and can interface with public databases for quantitative analysis. Biosensors currently remain in the laboratory research and validation phase, with sensor stability and reproducibility requiring further refinement. For instance, MIPWT exhibits approximately 30% adsorption capacity decline after five uses. Significant variations in peroxidase-like activity are observed between batches of CuS@Au-Pt preparations. Nevertheless, biosensors possess unique advantages, chiefly their multiplex detection capability. Through techniques such as fluorescence coding and multi-channel design, biosensors can simultaneously detect multiple toxins like BKA, OTA and AFB1. This eliminates the need for repeated sample processing and testing, substantially enhancing on-site screening efficiency, a capability difficult to achieve with traditional mass spectrometry. In summary, biosensors cannot replace gold standard methods such as mass spectrometry. Each has its appropriate application: biosensors are better suited for rapid on-site screening, while regulatory confirmation and absolute quantification still require large-scale instrumentation.

## 4. Reference of BKA Detection Technology Based on Structurally Similar Toxins

In recent years, increasing public concern regarding food safety and repeated incidents of BKA poisoning have intensified the demand for effective detection methods. As a toxin that has only recently gained attention due to frequent poisoning outbreaks, BKA has long been overlooked in research agendas, making it a typical emerging toxin requiring reliable detection strategies. Instrumental analysis methods for BKA detection currently primarily involve HPLC and LC–MS/MS. HRMS has further improved detection limits to as low as 0.01 μg/kg, providing high sensitivity and accuracy across diverse and complex sample matrices. Immunological techniques, including GICA, ic-ELISA, and TRFIA, are valued for their operational simplicity and rapid response, making them well-suited for on-site screening. Among these, TRFIA—using europium microsphere labels and a dual-probe quality control system—has substantially improved sensitivity, achieving a half-maximal inhibitory concentration (IC_50_) as low as 0.264 ng/mL. Biosensor technology integrates molecular recognition with signal transduction, leading to novel platforms such as MIP-PT-ESI-MS, dual-mode immunosensors, and core–shell nanozyme structures. These systems offer high sensitivity, with LOD reaching 0.05 μg/L, along with advantages such as multi-signal output and portability.

Despite considerable methodological diversity in available methodologies, several limitations persist. This study systematically retrieved and statistically analyzed relevant literature from databases including Web of Science, PubMed, and CNKI, as summarized in Table 2. Search terms encompassed “bongkrekic acid detection”, “BKA Biosensor”, and “BKA Immunoassay”, with a timeframe spanning from 1986 to July 2025. A total of 46 relevant detection studies were identified, comprising one publication from 1986 and 45 from the past decade. The selected literature encompasses three major technical approaches: instrumental analytical methods, immunological rapid methods, and biosensor rapid detection techniques. Inclusion criteria were full-text availability in English or Chinese, complete experimental data, and clear methodological descriptions. Exclusion criteria comprised studies solely addressing toxin toxicity mechanisms, lacking detection methodologies, or presenting insufficiently clear or incomplete data. First, the overall body of literature remains limited. As shown in Table 2, the total number of publications on the detection of BKA remains limited, with only 45 papers recorded from 2020 up to the present (2025). The table categorizes the literature by instrumental analytical methods, immunological rapid detection methods, and biosensor-based rapid detection techniques. The data reveal not only a low overall publication volume but also a lack of technological innovation in this field. Instrumental analytical methods still dominate, while the application of immunological and biosensor techniques, although increasing in recent years, remains relatively low in proportion. This reflects that innovative research on BKA detection is still sporadic, with insufficient technological accumulation and systematic optimization. Second, most methods still rely heavily on conventional technical pathways, showing insufficient integration of emerging interdisciplinary technologies. Third, there is a notable scarcity of recognition elements and signal amplification strategies specifically designed for BKA’s unique structural features, such as its polycarboxylate groups and long-chain unsaturated olefinic acids. In the exploration of novel detection technologies, structural analogs of BKA have recently attracted research interest. The latest immunoassays and biosensors targeting these analogs show considerable promise, offering new pathways for the effective detection of BKA. Fumonisin B1 (FB1), ochratoxin A (OTA), and aflatoxin B1 (AFB1) are widely recognized as the three major fungal toxins contaminating grains, oilseeds, and cereals. Each exhibits strong toxicity and high thermal stability. FB1 is produced by *Fusarium* species and is associated with health risks such as hepatocellular carcinoma and immunosuppression; OTA, generated by *Aspergillus* species, mainly causes nephrotoxicity and has potential carcinogenic effects; AFB1, produced by *Aspergillus flavus* and *A. parasiticus*, can lead to hepatocellular carcinoma after long-term low-dose exposure, as well as immunosuppression and impaired growth and development [89,90,91]. A detailed structural analysis of BKA, FB1, OTA, and AFB1 reveals significant similarities in three key aspects: molecular size, chemical properties and functional groups, and carbon chain architectures. First, this small size enables conjugation with carrier proteins such as bovine serum albumin (BSA) and ovalbumin (OVA) for the preparation of artificial antigens, which are widely employed in immunoassays, including ELISA and colloidal gold-based methods—further confirming their small-molecule nature. Second, regarding chemical properties and functional groups, all four toxins possess stable lipophilic structures. Polar functional groups within their molecules—such as hydroxyl, carboxyl, ester, and lactone groups—serve two important roles. On one hand, they allow chemical derivatization to enhance detection: modifications through these groups can significantly improve signal response and separation efficiency in instrumental analysis, suggesting the potential for cross-applicability of derivatization methods among different toxins. On the other hand, these groups facilitate affinity-based recognition: polar moieties such as hydroxyl and carboxyl groups act as key sites for specific molecular interactions (hydrogen bonding, electrostatic and hydrophobic interactions). For example, the multiple hydroxyl and ester groups in FB1; the hydroxyl and ester groups in OTA; the lactone ring in AFB1 (which can open under alkaline conditions to form derivatives with carboxyl and hydroxyl groups); and the hydroxyl and carboxyl groups in BKA all serve as antigenic epitopes or binding domains. This provides a molecular basis for developing highly selective immunoassays (ELISA and immunochromatography) and aptamer-based sensors. Third, regarding carbon chain structures, FB1, OTA, and AFB1 contain cyclic moieties (furan and coumarin rings), while BKA contains multiple double bonds forming a conjugated system. Similar to the cyclic conjugated systems in other toxins, this structure allows interactions with detection systems through ultraviolet absorption, fluorescence, or electrochemical activity—enabling the development of optical or electrochemical detection methods. These structural similarities not only offer insights into their toxicity mechanisms but also create opportunities for innovating rapid detection technologies for BKA by building upon the well-established rapid detection strategies used for FB1, OTA, and AFB1.

However, selecting an appropriate detection method depends not only on sensitivity but also requires comprehensive consideration of multiple factors, including application scenarios, cost, time, throughput, and on-site suitability. This paper systematically outlines the selection and recommendations for BKA detection technologies across different scenarios through the construction of Table 3. Concurrently, Table 4 presents the core performance parameters of various key detection technologies, providing researchers with more detailed data references. In terms of detection limits, instrumental methods for BKA, particularly HRMS, have achieved exceptional sensitivity, with limits of detection as low as 0.01 μg/kg. By comparison, immunological approaches such as TRFIA and biosensor platforms such as dual-mode immunosensors typically operate in the ng/mL range (0.264–8.4 ng/mL), suggesting room for further improvement relative to state-of-the-art detection platforms for FB1, OTA, and AFB1. For instance, Ag^+^-enhanced gold nanocluster strategies for OTA detection have reached an LOD of 0.003 μg/L (3 pg/mL), while CRISPR-Cas-based signal amplification for FB1 has achieved LODs as low as 0.45 pg/mL. Similarly, a nanozyme-based dual-mode sensor for AFB_1_ has demonstrated impressive sensitivity with an LOD of 0.0191 ng/mL. These benchmarks underscore the significant potential for advancing BKA rapid detection, particularly through the adoption of innovative signal amplification strategies. With respect to cross-reactivity profiles, BKA immunoassays exhibit both strengths and limitations. The use of high-affinity monoclonal antibodies enables exceptionally low cross-reactivity (<0.1%) with common mycotoxins such as aflatoxins and T-2 toxins, highlighting excellent selectivity. A notable challenge, however, is the significant cross-reactivity with its structural isomer, IBKA, which poses risks of quantitative inaccuracy or false positives in complex matrices. In contrast, antibodies or aptamers targeting FB1, OTA, and AFB1 often display moderate to high cross-reactivity with their structural analogs, such as FB2/FB3, OTB, and AFB2/AFG1/AFG2, respectively. Efforts to mitigate these issues are underway through sophisticated probe engineering and material design aimed at improving specificity. In summary, BKA detection technology, especially those aimed at point-of-care applications, can benefit substantially from the advanced strategies already established for FB1, OTA, and AFB1 detection. While current BKA antibodies show commendable specificity toward non-isomeric toxins, achieving discrimination from IBKA remains a critical hurdle. Future work should focus on refining recognition elements, such as developing highly specific nanobodies or aptamers. By leveraging successful methodologies used for structurally related toxins, BKA detection is well-positioned to achieve breakthroughs in sensitivity, specificity, and practical applicability. Recent innovations in rapid detection have focused predominantly on immunoassays and biosensor techniques. The following section reviews advances in the detection of FB1, OTA, and AFB1, with the aim of supporting the adaptation and application of such methods to BKA detection. This transfer of methodology is expected to enhance technical capabilities in monitoring food quality and safety.

### 4.1. Immunological Rapid Detection Methods

**Figure 5:**
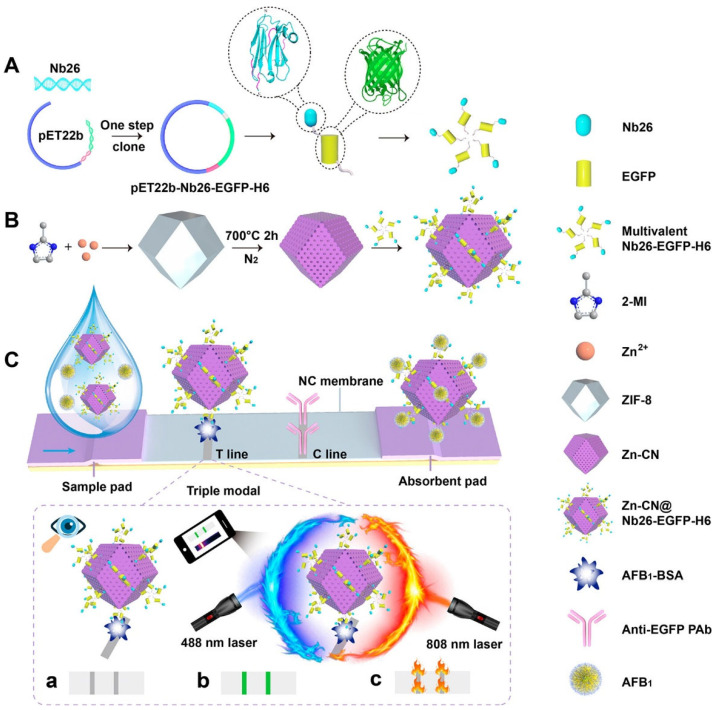
Construction of tLFIA Nanoplatform for AFB1. (**A**) Preparation of Self-Assembled Multivalent Fluorescent Nanobody Nb26-EGFP-H6. (**B**) Synthesis of Zn-CN@Nb26-EGFP-H6 Probe. (**C**) Competitive Mechanisms for Multimodal Analysis of AFB1. (**a**) Gray Scale Colorimetric Signal; (**b**) Fluorescent Signal; (**c**) Photothermal Signal [98].

Recent advances in immunological rapid detection methods have progressed along three main pathways: fundamental material innovation, biomolecular engineering optimization, and integrated system development, together forming a comprehensive technological chain. In the area of fundamental material innovation, Yu et al. [92] developed a polypyrrole nanoparticle (PPyNPs) and metal–phenolic resin composite probe (PMNPs) for the FB1 detection. This composite combines the broad-spectrum absorption properties of PPyNPs with the hydrophilicity and high antibody affinity of metal–phenolic resin (Au-TA structure). PMNPs act as highly efficient fluorescence quenchers, with a molar quenching coefficient 23.6 times higher than that of conventional gold nanoparticles, along with high stability in the presence of organic reagents and salt ions. This method achieved ultrasensitive detection, with a quantitative detection limit of 1.58 ng/mL. Jin et al. [93] functionalized dendritic mesoporous silica nanoparticles (DMSNs) for rapid FB1 detection. By introducing amino and thiol groups on the surface, they precisely controlled functional group density, significantly improving quantum dot (QD) loading efficiency and fluorescence retention. This approach addressed the issue of QD fluorescence quenching caused by surface defects in conventional carriers, providing a high-performance platform for probe construction. Chen et al. [94] reported Ag^+^-induced aggregation-induced emission enhancement (AIEE) in gold nanoclusters (GSH-AuNCs) and elucidated the underlying mechanism. Using NaBH_4_-reduced AgNP seeds (5 nm) as templates, they significantly enhanced the reduction in Ag^+^ by ascorbic acid (AA). This system attained a detection limit as low as 0.003 μg/L for OTA, establishing a solid foundation for subsequent immunoassays. Yang et al. [95] designed copper-doped MoOx nanoblossoms (CMO) as dual-mode signal probes for AFB1 detection. By incorporating copper into oxygen vacancies and encapsulating with tannic acid, they obtained highly water-soluble CMO nanomaterials capable of colorimetric and photothermal dual-mode output. The material exhibited high colorimetric intensity and a photothermal conversion efficiency of 45.58%, overcoming the sensitivity limitations of conventional gold nanoparticles (LOD = 0.0191 ng/mL). In biomolecular engineering optimization, Cai et al. [96] developed monovalent and divalent nanobodies fused with maltose-binding protein as FB1 antigen mimics, replacing conventional chemically synthesized antigens. This strategy improved nitrocellulose membrane immobilization efficiency while avoiding organic reagent contamination and batch variability. The method achieved a detection limit of 1.76 μg/kg in maize, with recovery rates ranging from 80.13% to 122.72%. Wu et al. [97] employed a SpyTag/SpyCatcher covalent binding system to program the assembly of nanobodies (Nb) with luciferases (Nluc/LuxSiti) on a 60-subunit protein nanostructure (SpyCatcher-mi3). By adjusting the Nb:Nluc ratio to 1:9, this system significantly enhanced signal intensity compared to conventional 1:1 fusion proteins. Using dual nanobody co-coupling, it enabled simultaneous detection of AFB1 and OTA, with a detection limit as low as 0.105 ng/mL for AFB1 and recovery rates between 91.5% and 101.4%. Xie et al. [98] genetically fused the anti-OTA nanobody (Nb28) with the large subunit of NanoLuc (LgBiT) to form an Lg-Nb fusion protein enabling precise 1:1 conjugation. This method addressed common issues in conventional homogeneous assays, such as fluorescence interference, loss of antibody activity due to chemical conjugation, batch variation, and by-product formation, offering a new model for homogeneous immunoassays for OTA. Li et al. [99] developed a genetically engineered, self-assembling multivalent fluorescent nanobody termed Nb26-EGFP-H6. This fusion protein combines a targeting nanobody, the enhanced green fluorescent protein (EGFP), and a histidine-rich peptide tag, which together drive its spontaneous assembly into multivalent nanostructures. The design provides intrinsic fluorescence without the need for external labeling. The construction of tLFIA Nanoplatform for AFB1 is shown in Figure 5. Through multivalent binding effects, the assembled nanobody shows a 16.5-fold increase in affinity and a 30-fold improvement in detection sensitivity relative to its monovalent counterpart. Moreover, the self-assembly approach overcomes the issues of steric hindrance and restricted orientation often encountered in traditional strategies for constructing multivalent antibodies. In integrated system development, Liu et al. [100] constructed a smartphone-based dual-mode detection device that synchronously integrates visible light (using AuNPs) and fluorescence (using TRFMs) lateral flow immunoassays. A custom Android app automatically recognizes test and control line signals, generates standard curves via RGB analysis, and quantifies results, allowing simultaneous detection of five mycotoxins: AFB1, ZEN, DON, T-2, and FB1. This system offers high-throughput multi-target detection and strong field applicability. Zeng et al. [101] established a phage-mediated digital PCR (dPCR) platform for ultrasensitive AFB1 detection. By combining magnetic bead-based competitive immunoassays with dPCR absolute quantification, they achieved a detection limit of 0.015 ng/mL—six times more sensitive than qPCR—with high consistency (r = 0.9847) compared to HPLC. Through the coordinated development and integration of these three stages, immunological rapid detection methods have established a complete technological framework that extends from fundamental probe design to end-user device applications. This progress has greatly advanced the detection technologies for FB1, OTA, and AFB1, leading to higher sensitivity, greater specificity, and improved ease of use.

### 4.2. Biosensor Rapid Detection Methods

**Figure 6:**
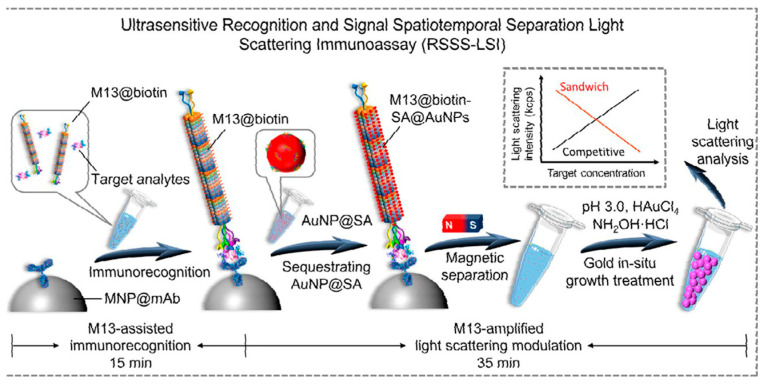
Designed M13 Phage-Assisted Recognition and Signal Spatiotemporal Separation Light Scattering Immunoassay Strategy for Ultrasensitive Quantitative Detection of Low-Abundance Target Analytes [107].

**Figure 7:**
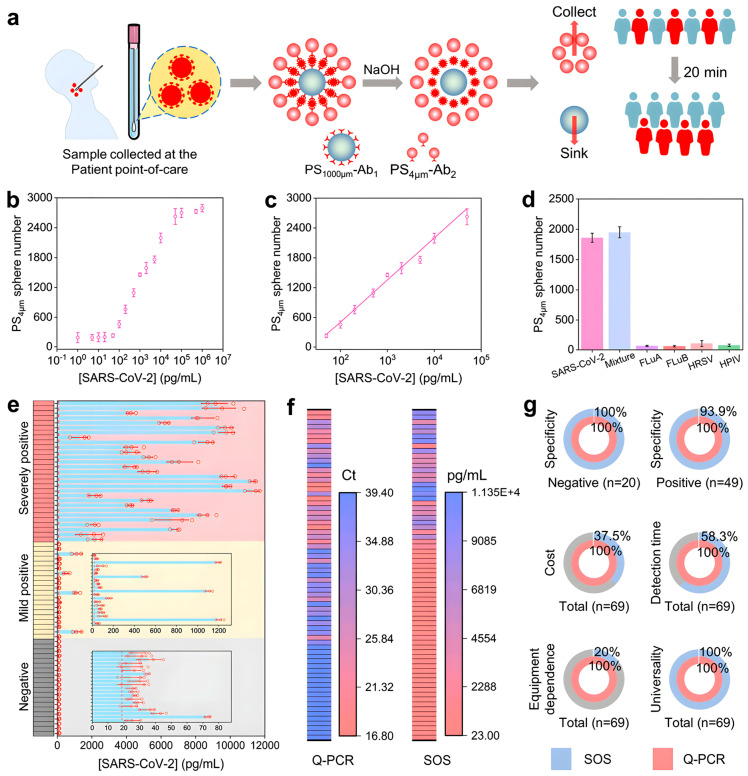
SOS platform for SARS-CoV-2 detection. (**a**) Schematic of the SOS platform for detection of SARS-CoV-2. (**b**) Standard curve. (**c**) Linear regression of the concentration measured by the SOS platform. (**d**) The specificity test was performed by setting FLuA, FLuB, HRSV, and HPIV as controls. (**e**) Quantitative measurements of SARS-CoV-2 in clinical samples using the SOS platform with the clinical diagnosis of each patient. Bar graph data represent mean ± SD of replicates (*n* = 3 biologically independent experiments); each red circle represents the result from one experiment, and the dashed line indicates the LOD of the SOS platform. (**f**) Cluster map produced from results measured by the SOS platform and Q-PCR for 69 eligible (including positive and negative samples) patients. (**g**) SARS-CoV-2 detection accuracy, cost, time, equipment dependence, and universality for the SOS platform in different patient groups using Q-PCR as a reference (negative, *n* = 20; positive, *n* = 49) [111].

Innovations in biosensor rapid detection Methods primarily focus on three aspects: nanomaterial design, signal amplification strategies, and multi-modal integration. In nanomaterial design, the use of materials with unique physicochemical properties has led to the development of highly sensitive and specific nanomaterials that significantly enhance signal intensity and improve sensor performance. Behnaz Naghshbandi et al. [102] developed an electrochemical aptasensor based on screen-printed carbon electrodes (SPCE) modified with AuNPs for FB1 detection, achieving a LOD of 0.14 ng/mL. The study employed electrodeposition to uniformly coat AuNPs onto the SPCE surface, which improved electrode conductivity and specific surface area. Li et al. [103] synthesized Pt NPs/Fe-MOG composites by combining the porous structure of Fe-MOG with the high catalytic activity of Pt nanoparticles to enhance peroxidase-like activity. Kornautchaya Veenuttranon et al. [104] introduced a nanocomposite composed of nickel oxide (NiO) and carboxylated multi-walled carbon nanotubes (MWCNT-COOH). This design utilizes the abundant active sites of NiO nanosheets and the high conductivity and large surface area of MWCNT-COOH to achieve ultrasensitive detection of OTA and ZEN, with LODs as low as 6 ng/mL and 15 ng/mL, respectively. Niu et al. [105] designed a two-dimensional covalent organic framework (TA-P COFs) film grown directly on an electrode surface under mild conditions for detecting AFB1. This method addresses issues of poor adhesion and uncontrolled size seen in traditional COF powders. The material shows high stability, low biotoxicity, and an ordered pore structure that facilitates charge transfer, thereby improving photoelectrochemical conversion efficiency. In signal amplification strategies, techniques such as enzyme catalysis and chemiluminescence are used to amplify weak signals from biomolecular interactions, thus lowering detection limits. Li et al. combined magnetic separation with peroxynitrite-like catalytic amplification. Using the magnetic properties of Fe-MOG for rapid separation and PtNPs to catalyze the TMB colorimetric reaction, this method produced strong color changes and high catalytic efficiency, greatly enhancing the sensitivity for FB1 detection (LOD = 2.7 pg/mL). In another approach, Li et al. [106] utilized a dual-enzyme cyclic amplification technique for FB1 detection, which involves T7 exonuclease cycling (releasing activated DNA to trigger the CRISPR/Cas12a system) and Cas12a trans-cleavage (non-specifically cleaving ssDNA-FAM to release fluorescent signals). The combination of dual-enzyme amplification and CRISPR technology achieved ultra-high sensitivity, with a detection limit of 0.45 pg/mL. As detailed in Figure 6, Fang et al. [107] integrated immunorecognition (spatiotemporal separation) with gold in situ growth (GISG), along with a biotin–streptavidin cascade system, to amplify signals for OTA detection. In this method, the M13 phage acts as a lever: one end binds the target, while the other captures numerous gold nanoparticle seeds (AuNP@SA) via biotin–streptavidin interaction. The GISG amplification involves free AuNP@SA seeds reducing chloroauric acid with hydroxylamine, leading to particle growth from 8 nm to 200 nm. This approach improved detection sensitivity by approximately four orders of magnitude compared to conventional phage-based ELISA. Sareh Sadat Moshirian-Farahi et al. [108] utilized a nanozyme-confined effect for signal amplification in AFB1 detection. By confining the catalytic reaction within cellulose nanofiber (NF) pores, the TMB oxidation products (blue signal) were concentrated, resulting in a sevenfold increase in sensitivity compared to open systems and a detection limit as low as 3.9 pg/mL—two to three orders of magnitude lower than traditional colorimetric methods. In multi-modal integration design, it combines various detection techniques—such as electrochemical, optical, and surface plasmon resonance—to leverage their complementary advantages and achieve multidimensional toxin analysis. Neelakandan M et al. [109] used a statistical method (principal component analysis, PCA) combined with surface-enhanced Raman spectroscopy (SERS) fingerprinting for rapid FB1 detection. By applying PCA to reduce the dimensionality and classify mycotoxin Raman spectra, all SERS profiles were clearly grouped into four distinct clusters. This approach successfully differentiated four mycotoxins (AFB_1_, ZEN, ALT, and FB_1_) with a detection limit as low as 0.45 pg/mL, making the method suitable for high-throughput screening of complex samples. Wang et al. [110] developed a fully integrated point-of-care testing (POCT) system for OTA detection, which combined microfluidic fabrication, single-particle-level biosensing, and portable device design to bridge the gap between laboratory and field applications. Key innovations encompass a six-channel microfluidic chip, a 3D-printed integrated device, and magnetic functional designs. The microfluidic chip enables the fabrication of uniform multi-chamber microspheres with precisely controlled particle sizes. The 3D-printed device integrates 37 °C temperature control, multi-wavelength excitation light sources, optical filters, and a mobile imaging module to achieve one-button on-site detection. Magnetic compartments guarantee consistent imaging orientation of microspheres, while a magnetic alignment design addresses challenges in spatial signal decoding. Lu et al. [111] applied a portable Coulter counter to simultaneously quantify the count and size of multi-scale microspheres, enabling triple-target detection of multiple toxins (including AFB1) and viruses. The SOS platform for SARS-CoV-2 detection is illustrated in Figure 7. This low-cost strategy is particularly suitable for use in resource-limited settings. Through coordinated progress in these three areas: nanomaterial design, signal amplification, and multimodal integration. Biosensor-based rapid detection methods have established a comprehensive technological framework that significantly improves the sensitivity, specificity, and practicality of detecting FB_1_, OTA, and AFB_1_, driving considerable advances in the field.

## 5. Conclusions and Future Perspectives

The detection of BKA, a key virulence factor from *Burkholderia gladioli*, threatens public health. While recent technological breakthroughs have greatly enhanced detection technologies, instrumental analyses, such as HRMS, which provide benchmark sensitivity (LOD~0.01 μg/kg), are inherently limited by their cost, technical demands, and lack of field-deployability. Within immunological methods, the time-resolved fluorescence immunoassay (TRFIA) utilizing europium nanoparticle labels and a dual-probe quality control system has achieved a linear detection range of 0.1–10 ng/mL. Meanwhile, colloidal gold immunochromatographic assays offer the advantage of visual, qualitative on-site screening. Nevertheless, immunological techniques in general are still limited by antibody specificity and stability, as well as the potential for false-positive results. Biosensing technology has demonstrated considerable potential for multimodal integration. For instance, dual-mode immunosensors combining fluorescent and colorimetric signals enable mutual verification, achieving a detection limit as low as 5.7 ng/mL. Similarly, sensors utilizing core–shell biomimetic nanospheres exploit enzyme-like activity to amplify signals, lowering the limit of quantification to 0.66 ng/mL. These advances offer promising avenues for on-site rapid detection; however, their practical application remains limited, and issues regarding long-term stability need to be addressed.

Notably, BKA shares important structural and chemical characteristics with mycotoxins such as OTA, FB1, and AFB1, including polarity, key functional groups, and carbon-chain architecture. These compounds are all small molecules (with molecular weight below 1000 Da) exhibiting lipophilicity and thermal stability and contain polar groups such as hydroxyl and carboxyl groups as well as conjugated double bonds. These shared characteristics enable the transferability of detection mechanisms, such as antigen–antibody binding, nanomaterial adsorption, and optical/electrochemical responses, laying a solid theoretical foundation for the cross-application of detection technologies. Specifically, cutting-edge strategies developed for other mycotoxins offer directly transferable solutions for BKA detection. In immunoassays, the mono-/bivalent nanobody technology employed in FB1 detection could enhance the immobilization efficiency of BKA antigen mimics while reducing organic solvent contamination. In biosensing, enzymatic cycling amplification using CRISPR-Cas systems for aflatoxin and in situ growth-mediated signal amplification of gold nanoparticles for ochratoxin show potential to lower the detection limit of BKA by 1–2 orders of magnitude. Furthermore, multimodal integrated platforms, such as smartphone-driven dual-mode detection devices, could enable high-throughput, on-site quantification of BKA, overcoming the limitations of conventional laboratory-based methods.

A major forthcoming direction is the development of integrated sensing platforms that deliver both high sensitivity and rapid, on-site operation. Achieving this goal will require the synergistic integration of functional nanomaterials, enhanced signal amplification strategies, and portable instrumentation. Such integrated systems are critical to translating detection capabilities into practical tools for mitigating BKA poisoning risks.

## Figures and Tables

**Table 1:** Globally reported cases of BKA poisoning.

Location		Time	Number of Poisoning	Death	Reference
China	Zhejiang	2018	3	1	[11]
	Guangdong	2020	11	1	[12]
	Heilongjiang	2020	9	9	[13]
	Henan	2023	2	1	
	Taiwan	2024	33	2	[14]
	Jiangxi	2025	1	0	
	Hubei	2025	1	0	[15]
Indonesia	java	1895	9	5	[16]
		1951–1975	7216	850	
		1975	1036	125	[17]
		1977	400	70	[18]
		1983	450	42	[19]
		1988	200	14	[20]
Mozambique	Tete	2015	234	75	[21]
North America		2024	2	1	[22]

**Table 2:** The total number of publications on the detection of BKA.

Time Period	Instrumental Analytical Methods	Immunological Rapid Detection Methods	Biosensor Rapid Detection Techniques	Annual Total
2020 and earlier	6	2	0	8
2021	6	1	0	7
2022	4	1	1	6
2023	6	1	2	9
2024	4	3	3	10
2025 (to date)	4	1	1	6
Classification Total	29	9	7	46

**Table 3:** Application Scenarios and Technology Recommendations for BKA Detection.

Core Application Scenario	Recommended Detection Technology (ies)	Justification for Recommendation	Reference
Laboratory-based Trace & Accurate Detection (Regulatory)	High-Resolution Mass Spectrometry (HRMS, Q-Orbitrap)	Highest sensitivity (LOD down to 0.01 µg/kg), accurate qualitative and quantitative capability, suitable for multi-matrix trace analysis, meeting stringent regulatory and research requirements.	[59,69]
On-Site Rapid Screening (Enforcement/Primary-Level Testing)	Time-Resolved Fluoroimmunochromatographic Assay (TRFIA), Dual-Mode Immunosensor (Fluorometric/Colorimetric), Colloidal Gold Immunochromatographic Assay (CGIA)	Simple operation (<30 min), no need for complex instrumentation, supports visual qualitative or portable device quantitative readout, fitting the need for rapid on-site response.	[73,74,80]
Clinical Sample Testing (Poisoning Diagnosis)	Stable Isotope Dilution—LC-MS/MS, UPLC-MS/MS (for plasma/urine)	Adapted to biological matrices (plasma/urine), sensitivity reaches 0.02 µg/L level, fills the technical gap for clinical diagnosis.	[60,62]
High-Throughput Batch Testing (Large-Scale Sampling)	CS-AuNPs Dual-Mode Platform (96-well plate), Flow Cytometry Fluoroimmunoassay	Supports simultaneous testing of batch samples, high throughput and efficiency, suitable for large-scale sampling inspection scenarios.	[82,84]
Low-Cost Primary Screening (Community)	Colloidal Gold Immunochromatographic Assay (CGIA), Smartphone Colorimetric Method (CS-AuNPs)	Low cost, no professional expertise required for operation, methods allow visual judgment, suitable for preliminary screening in resource-limited primary settings.	[70,82]

**Table 4:** Key Parameters of BKA Detection Technologies.

Method Category	Specific Technique	LOD	Analysis Time	Cost	Throughput	On-Site Suitability	Reference
Instrumental Analysis	HPLC (GB Standard)	1.0 μg/kg	Long (Hours)	High	Low	No	[55]
	HPLC-MS/MS	0.02–1.0 μg/kg	Medium–High–	Very High	Medium	No	[60]
	UPLC-MS/MS	0.1 μg/kg	Medium	Very High	Medium	No	[59]
	HRMS (Q-Orbitrap)	0.01 μg/kg	Medium	Extremely High	Medium	No	[69]
Immunological Methods	Colloidal Gold Immunoassay (CGIA)	1.2 μg/kg	Fast (<15 min)	Low	Medium	Yes	[70]
	Indirect Competitive ELISA (ic-ELISA)	0.99 μg/L (ng/mL)	Medium (1–2 h)	Low–Medium	Medium	Limited	[71]
	Time-Resolved Fluoroimmunoassay (TRFIA)	0.5 μg/kg	Fast (<10 min)	Low–Medium	Medium–High	Yes	[73,74]
Biosensors	Dual-Mode Immunosensor (Fluoro/Colorimetric)	5.7–8.4 μg/L (ng/mL)	Fast (<30 min)	Low-Medium	Medium	Yes	[80]
	CS-AuNPs (UV-Vis/Smartphone)	~0.006 μg/L (3.43 nmol/L)	Fast (<30 min)	Low	High (96-well)	Yes	[82]
	Core–Shell Nanozyme LFIA (CAP-NLFIA)	0.5 μg/L (ng/mL)	Fast (~15 min)	Low–Medium	Medium	Yes	[83]
	Flow Cytometry Fluoroimmunoassay	0.56 μg/kg	Fast (<30 min)	Medium	High	Limited	[84]
	MIP Wooden-Tip ESI-MS	0.05 μg/L	Fast (<30 min)	Low	Medium	Yes	[75]

## Data Availability

The original contributions presented in this study are included in the article. Further inquiries can be directed to the corresponding authors.
